# Data on the acceptance of a tourism navigation system applying structuring equation modeling analysis

**DOI:** 10.1016/j.dib.2018.09.002

**Published:** 2018-09-06

**Authors:** Huei-Ming Chiao, Yu-Li Chen, Wei-Hsin Huang

**Affiliations:** a Graduate Institute of Design Science, Tatung University, Taipei, Taiwan, ROC; b Department of Digital Game and Animation Design, Taipei University of Maritime Technology, Taipei, Taiwan, ROC; c Graduate Institute of International Tourism and MICE Industry, Department of Applied Foreign Languages, Lunghwa University of Science and Technology, Taoyuan, Taiwan, ROC; d Department of Media Design, Tatung University, Taipei, Taiwan, ROC

## Abstract

The data presented in this article relate to the acceptance of an online tourism search technology by students from a Science and Technology University in Taiwan. The data were collected using quasi-experiment research design and a survey questionnaire. A structural equation modeling analysis was employed for data analysis using AMOS statistical software. For further study findings and interpretation, please refer to the research article entitled “Examining the Usability of an Online Virtual Tour-Guiding System for Cultural Tourism Education” (Chiao et al., 2018).

**Specification table**

**Table:** 

**Subject area**	Social Science; Information and Communicative Technology
**More specific subject area**	Tourism education; multimedia science, virtual reality; game-based learning
**Type of data**	Table, image
**How data was acquired**	Survey
**Data format**	Raw and analyzed data
**Experimental factors**	Seven factors are included in the predicted model of tourism navigation system acceptance: five exogenous variables and 2 endogenous variables. The accuracy of data input, missing observations and outliers, as well as internal consistency reliabilities for all major variables were treated before conducting the structural equation modeling analysis.
**Experimental features**	A confirmatory factor analysis and a structural equation modeling analysis were conducted for the model.
**Data source location**	Taoyuan, Taiwan
**Data accessibility**	With this article
**Related research article**	[1] H.-M. Chiao, Y.-L. Chen, W.-H. Huang, Examining the usability of an online virtual tour-guiding platform for cultural tourism education. Journal of Hospitality, Leisure, Sport & Tourism Education, 23, 2018, 29–38.

**Value of the data**

• The dataset is beneficial for identifying and understanding valuable opportunities in virtual reality and game-based learning research as well as interdisciplinary research on tourism and multimedia game science.
• In addition to the four exogenous variables in the UTAUT model of Venkatesh et al. [2], Interaction was added due to the feature of the platform. Data can be compared with other studies either using the same construct or adding another variable as an exogenous or moderating variable.
• This dataset can be used as a benchmark to compare learning effectiveness and technology acceptance and further pedagogical approaches and technological innovations can be designed and tested.

## 1 Data

The data file spreadsheet accompanying this article consists of 391 rows and 27 columns of data. Each row represents an individual student response for a questionnaire. A 7-point Likert scale was used to allow the learner to indicate how much they agree or disagree with a particular statement, so a numerical value in the data file means the learner׳s level of agreement, with 7 being strongly agree and 1 being strongly disagree.

Each questionnaire item in the columns was given a label as shown in the first row. Per is the short form for Performance expectance; SoI for Social Influence; Eff for Effort Expectance; FaE for Facilitating Environment; Int for Interaction; ItU for Intention to Use; and Beh for Behavioral Use. After filtering of the data and through a reliability test, six items of Performance Expectance remained for a structural equation modeling analysis: Per01, Per02, Per03, Per04, Per05 and Per 07; four items of Effort Expectance: Eff02, Eff04, Eff,05 and Eff,06; three items of Social Influence: SoI01, SoI03 and SoI04; four items of Facilitating Environment: FaE01, FaE02, FaE03 and FaE04; three items of Interaction: Int01, Int02 and Int03; four items of Intention to Use: ItU01, ItU02, ItU03 and ItU04; and three items of Behavioral Use: Beh01, Beh02 and Beh03 (see Table 1).

**Table 1:** The data file items.

**Factors**	**Items remained after reliability testing**
Performance expectance	Per01, Per02, Per03, Per04, Per05, Per07
Effort expectance	Eff02, Eff04, Eff05, Eff06
Social influence	SoI01, SoI03, SoI04
Facilitating environment	FaE01, FaE02, FaE03, FaE04
Interaction	Int01, Int02, Int03
Intention to use	ItU01, ItU02, ItU03, ItU04
Behavioral use	Beh01, Beh02, Beh03

## 2 Experimental design, materials, and methods

**Fig. 1:**
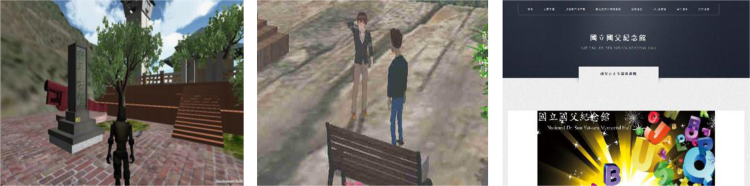
The online tourism navigation system created by the authors.

The development of the integration of information and communication technology services is an inevitable trend for the future of the tourism industry׳s economic development. The researchers created an online navigation system with a 3D virtual reality and game-based environment using Unity software (see Fig. 1). The tourism navigation system acceptance data were collected from the students of the College of Humanities and Design at a science and technology university in Taiwan. These data were provided in a Microsoft Excel Worksheet as supplementary data for this article. They were used for a structural equation modeling analysis to examine student perception and acceptance of the navigation system.

The data were obtained from a quasi-experiment. After student learning in the designed tourism navigation system, data about student perception and actual use of the system were collected by using a web-based questionnaire which was developed based on Venkatesh, Morris, Davis, and Davis’ the Unified Theory of Acceptance and Use of Technology (UTAUT) model [2]. Four variables in the UTAUT model were included as exogenous variables to predict student behavioral use of the tourism navigation system. These are Performance Expectance (Per), Effort Expectance (Eff), Social Influence (SoI), and Facilitating Environment (FaE). As the navigation system features interaction between learners and the system, Interaction (Int) variable was added as another exogenous variable of the predicted model. Intention to Use (ItU) and Behavioral Use (Beh) are the two endogenous variables.

The common steps for executing a structural equation modeling (SEM) analysis are as following: specification, identification, evaluation and modification of the model. Data were initially screened and treated for the issues of accuracy of data input, missing observations and outliers. Data were examined for the concurrent, convergent, and discriminant validities by a correlation matrix. A confirmatory factor analysis (CFA) was conducted to obtain the internal consistency reliabilities for all major variables. After CFA, one item was removed from Performance Expectance, Social Influence, and Facilitating Environment respectively and two items were removed from Effort Expectance. The Cronbach׳s Alpha coefficient values of all major variables range from 0.85 to 0.935.

Before the experiment, a hypothesized model was established in that all five exogenous variables were hypothesized to be positively related to the endogenous variable, Behavioral Use. Performance Expectance, Effort Expectance, Social Influence, and Interaction were hypothesized to have direct effects on Intention to Use, which has a direct effect on Behavioral Use. That is, Intention to Use mediates the effects of Performance Expectance, Effort Expectance, Social Influence, and Interaction on Behavioral Use. Facilitating Environment was hypothesized to have a direct effect on Behavioral Use.

The *AMOS* statistical software was used for the data analysis. Structural equation modeling analysis comprises the evaluation of the measurement model and that of the path model. The evaluation of the goodness-of-fit of the model consists of several types of model fit indices: absolute fit indices, relative fit indices, parsimony fit indices, and those based on the noncentrality parameter [3].

The various types of the above-mentioned good-of-fit indices are shown on Table 2. Each type has several fit indices, which inform the different aspects of evaluation. The full names of these indices are spelled out on the right column in the Table 2.

**Table 2:** The overall model fit measures.

**Types of indices**	**Fit indices**	**Full name**
	$\chi_{\left(308\right)}^{2}$	
	GFI	Goodness of fit index
	AGFI	Adjusted Goodness of fit index
**Absolute fit measures**	RMR	The root mean square residual
	SRMR	The standardized root mean square residual
	RMSEA	The root mean square error of approximation
	NFI	Normed Fit Index
	NNFI	Non-Normed Fit Index
	CFI	Comparative Fit Index
**Comparative fit measures**	IFI	Incremental Fit Index
	RFI	Relative Fit Index
	TLI	Tucker-Lewis index
	PNFI	Parsimonious Normed Fit Index
**Parsimonious fit measures**	PGFI	Parsimonious Goodness-of-Fit Index
	CN	Hoelter׳s Critical N

The suggested guidelines and criteria for goodness-of-fit of a model are shown in Table 3. In general, if the vast majority of the indices indicate a good fit, then there probably is a good fit [4]. During the process of conducting the structural equation modeling analysis using this dataset via Amos statistical software, the predicted model was continuously assessed for the goodness-of-fit based on the model fit indices and suggested guidelines shown on Table 3. Please refer to the related research study for the value of the resulting model.

**Table 3:** Preliminary, overall, and comparative model fit indices.

	**Model fit indices**	**Suggested guidelines**	**Model fit**
Preliminary fit criteria	error variances	Positive	Yes
	error variances	Significance	Yes
	standard error of estimate	Smaller, better	Yes
	standardized estimates	Between 0.50 and 0.95	Yes
Overall model fit criteria	χ^2^ value	ns	ns
	RMSEA	<0.08 or 0.10	Yes
	RMR	<0.05 or <0.1	Yes
	SRMR	<0.05	Yes
Comparative fit criteria	NFI	>0.90	Yes
	NNFI	>0.90	Yes
	CFI	>0.90	Yes
	IFI	>0.90	Yes
	RFI	>0.90	Yes
	TLI	>0.90	Yes

*Note*. Refs. [5], [6], [7], [8].

The structural equation modeling analysis using the dataset reveals a model fit for the tourism navigation system learning and acceptance. For the interpretation of the causal paths of the structural model, please refer to the related research study.
