# Supplementary material for: Data on the acceptance of a tourism navigation system applying structuring equation modeling analysis
Source: Data Brief. 2018 Sep 6;20:1392–6. doi: 10.1016/j.dib.2018.09.002 (PMC6148738; doi:10.1016/j.dib.2018.09.002)
Supplement: Supplementary file 1 — Supplementary material [file mmc1.docx]

**Conflict of Interest**

The authors have no affiliation with any organization with a direct or indirect financial interest in the subject matter discussed in the manuscript.
